# Effects of real-time remote cardiac rehabilitation on exercise capacity and quality of life: a quasi-randomised controlled trial

**DOI:** 10.1186/s12877-023-04113-8

**Published:** 2023-06-24

**Authors:** Yoshitatsu Fukuta, Shinichi Arizono, Shinichiro Tanaka, Tomonori Kawaguchi, Natsumi Tsugita, Takahiro Fuseya, Junichi Magata, Yuichi Tawara, Tomonori Segawa

**Affiliations:** 1 Department of Rehabilitation, Asahi University Hospital, 3-23 Hashimoto, Gifu, Gifu 500-8523 Japan; 2 School of Health Sciences, Seirei Christopher University, Hamamatsu, Shizuoka Japan; 3 Department of Cardiology, Asahi University Hospital, Gifu, Gifu Japan

**Keywords:** Remote cardiac rehabilitation, World Health Organization Disability Assessment Schedule, Peak oxygen uptake

## Abstract

**Background:**

The impact of real-time remote cardiac rehabilitation (CR) on health and disability-related outcomes and its correlation with physical function are unknown. We compared the effectiveness of real-time remote CR with that of hospital-based CR on physical function improvement and physical functions of improvement (Δ) to clarify the relationship between health and disability at baseline.

**Methods:**

Patients with cardiovascular diseases (CVDs) were enrolled (*n* = 38) in this quasi-randomised controlled trial and underwent 4 weeks of hospital-based CR, followed by 12 weeks of remote or hospital-based CR based on quasi-randomised allocation. Patients were assessed at baseline and after 12 weeks of remote or hospital-based CR using the shortened version of the World Health Organization (WHO) Quality of Life scale (WHOQOL-BREF) for subjective satisfaction, WHO Disability Assessment Schedule (WHODAS2.0-J) for objective performance, and cardiopulmonary exercise test for physical function and peak oxygen uptake (peak VO_2_). The trends in measured variables from baseline to the post-CR stage were analysed.

**Results:**

Sixteen patients (mean age, 72.2 ± 10.4 years) completed remote CR, and 15 patients (mean age, 77.3 ± 4.8 years) completed hospital-based CR. The post-CR physical function differed significantly between the groups (Δ_peak_ VO_2_, 2.8 ± 3.0 versus 0.84 ± 1.8 mL·min^−1^·kg^−1^; *p* < 0.05). The differences in post-CR changes in the WHOQOL-BREF scores between the groups were insignificant. The post-CR changes in the WHODAS2.0-J scores were significantly lower in the remote CR group than in the hospital-based CR group (ΔWHODAS2.0-J score, –8.56 ± 14.2 versus 2.14 ± 7.6; *p* < 0.01). Forward multiple stepwise regression analysis using overall data showed that the intervention method (β = 0.339, *p* < 0.05), baseline cognition (β =  − 0.424, *p* < 0.05), and social interaction level (β = 0.658, *p* < 0.01; WHODAS2.0-J) were significant independent contributors to Δpeak VO_2_ (r^2^ = 0.48, F = 8.13, *p* < 0.01).

**Conclusions:**

Remote CR considerably improved physical function and objective performance in patients with CVDs. Remote CR can be used to effectively treat stable patients who cannot visit hospitals.

**Trial registration:**

This interventional trial was registered at the UMIN-CTR registry (trial title: Development of remote programme for cardiac rehabilitation using wearable electrocardiograph; trial ID: UMIN000041746; trial URL: https://center6.umin.ac.jp/cgi-open-bin/ctr_e/ctr_view.cgi?recptno=R000046564; registration date: 2020/09/09).

## Background

Currently, Japan has a major health economic burden, of which approximately 20% is attributable to cardiovascular diseases [1]. Cardiac rehabilitation (CR) is a non-pharmacological method for reducing mortality and rehospitalisation rates and improving physical function [1–4]. The Japanese medical insurance system provides CR coverage for 150 days, and CR is a cost-effective treatment [5, 6]. Therefore, continuous CR is a crucial step that lowers treatment costs and reduces mortality and readmission rates. However, there is a shortage of medical personnel in rural areas, which poses a major challenge to the medical care delivery system [7]. Remote medical care can help solve such challenges. In particular, techniques for remote and continuous monitoring of vital signs, such as electrocardiography, are critical for improving the detection rates of arrhythmia while improving accessibility for older individuals during exercises. However, the current remote medical system is inadequate.

CR is a comprehensive intervention that includes exercises, nutritional management, and education. Convalescence CR is commonly employed among outpatients, but it has a low continuity rate [8]. Moreover, the CR participation rates in Japan are lower than those in other countries [9], and this is attributed to the distance between patient housing and medical centres as well as the mobility restrictions of older adults. Real-time remote CR can serve as an effective solution for overcoming distance and mobility limitations. Remote CR has been conducted for non-monitoring purposes, and few real-time monitoring studies exist [10, 11]. Real-time monitoring methods improve physical function [12], while non-monitoring CR may improve quality of life [13].

The Japanese version of the World Health Organization Quality of Life Instrument (WHOQOL-BREF) and Japanese version of the World Health Organization Disability Assessment Schedule 36-item self-administered (WHODAS2.0-J) are two of the measurement tools used to assess quality of life and disability, respectively.

WHOQOL-BREF assesses subjective satisfaction, and WHODAS2.0-J assesses objective performance. WHODAS2.0-J asks what a person “does” in a particular domain, while WHOQOL-BREF asks what the person “feels” in that domain.

However, research on subjective and objective satisfaction using real-time monitoring remote CR is scarce, and there are no reports on the association of subjective and objective satisfaction with physical functioning using remote CR. Furthermore, physical functions, subjective satisfaction, and objective performance may be adversely affected during the coronavirus disease 2019 (COVID-19) pandemic, and mental health aspects need to be considered. The development of remote CR protocols can play a pivotal role during the COVID-19 pandemic. Therefore, the purpose of this study was to determine whether subjective satisfaction, objective performance (assessment of activity limitations and participation restrictions), and physical function are comparable (non-inferiority) for real-time CR and hospital-based CR.

In addition, we determined the relationship between baseline and CR physical function. We hypothesised that real-time remote CR is equivalent to hospital CR in terms of subjective satisfaction and objective performance. We also evaluated the feasibility of and challenges in real-time remote CR.

## Methods

### Study design

This pragmatic, parallel-group, non-inferiority, pilot quasi-randomised controlled trial was conducted from September 2020 to April 2022, and 40 patients with cardiovascular diseases (CVDs) were shortlisted. Initially, hospital-based CR was performed for 4 weeks for all participants. Then, quasi-randomised allocation was performed, followed by 12 weeks of remote or hospital-based CR. Intergroup comparisons were performed. The differences between the two groups were evaluated. In addition, each group underwent baseline and 12-week assessments for each comparison. Patients were excluded from the study if any exclusion criterion was met during the 4-week hospital CR and cardiopulmonary stress test.

Hospital-based CR and remote CR exercises were based on the American College of Sports Medicine [14] and the guidelines of the Japanese Society of Cardiology [15].

### Inclusion and exclusion criteria

Patients with cardiovascular disease who agreed to provide consent for the study were included. Patients with unstable symptoms, those living alone, and those with arrhythmia who met the criteria for treatment discontinuation were excluded. In addition, all patients were trained to use the ECG monitoring equipment and were excluded if they had difficulty using it appropriately. Furthermore, patients who did not have a suitable means of communication (telephone or internet) at home were excluded.

### Cardiopulmonary exercise test (CPX)

CPX was performed to measure VT and % peak VO_2_. CPX was performed using a cycle ergometer (STB-3400, NIHON KOHDEN, Tokyo, Japan). VO_2_, carbon dioxide production, and minute ventilation were measured using an expired gas analyser (AE-310 MINATO, Tokyo, Japan). Heart rate was continuously monitored throughout the test using the stress system STS 2100 (NIHON KOHDEN). A ramp stress test was used to determine the stress system for CPX.

The ventilatory threshold was determined using the ventilatory equivalent method [16]. The intensity of this activity causes the first rise in the ventilatory equivalent of oxygen without a concurrent increase in the ventilatory equivalent of carbon dioxide [16].

### Health and disability

Subjective satisfaction was measured using the WHOQOL-BREF [17]. Its reliability and validity have been reported [18]. The World Health Organization Quality of Life (WHO-QOL) is used for assessing quality of life. The short version of the WHO-QOL is the WHOQOL-BREF, which consists of 26 questions. Among them, 24 questions are categorised into four domains: physical health with seven items, psychological health with six items, social relationships with three items, and environment area with eight items. The two remaining questions assess the domains of the perception of quality of life and health of the patients. The total score of each domain was calculated according to the score table guidelines provided by the WHO in the original WHOQOL-BREF [19]. An increase in the WHOQOL-BREF score is considered an improvement.

Objective performance was measured using the WHODAS2.0-J. The WHODAS2.0-J is a reliable and valid instrument for the assessment of function among the older Japanese population [20]. WHODAS-2 is commonly used as an international and interdisciplinary means of measuring disability. Furthermore, it is the only measurement tool based on the International Classification of Functioning, Disability and Health biopsychosocial model [21].

The WHOQOL-BREF is used to measure subjective well-being based on patient satisfaction with performing routine activities, while the WHODAS2.0-J is used to measure the feasibility of performing activities of daily living in terms of physical function [22]. The WHODAS2.0-J assesses the activity limitations and participation restrictions experienced by an individual. A decrease in the WHODAS2.0-J score is considered an improvement.

### Physical function

Physical function was measured using peak VO_2_, VT, leg strength, 6-min walk test, and grip strength. The primary outcome was physical function, whereas the secondary endpoints were health and disability.

The participants were informed of the details of the study, and they provided written consent to participate before enrolment. The study protocol followed the Declaration of Helsinki and its later amendments or comparable ethical standards. The study protocol was approved by the Institutional Review Boards of Asahi University Hospital (approval number: 2020–04-05) and Seirei Christopher University (approval number: 21–046-01).

### Real-time remote CR programme and electrocardiogram (ECG) monitoring system (Fig. 1)

**Fig. 1:**
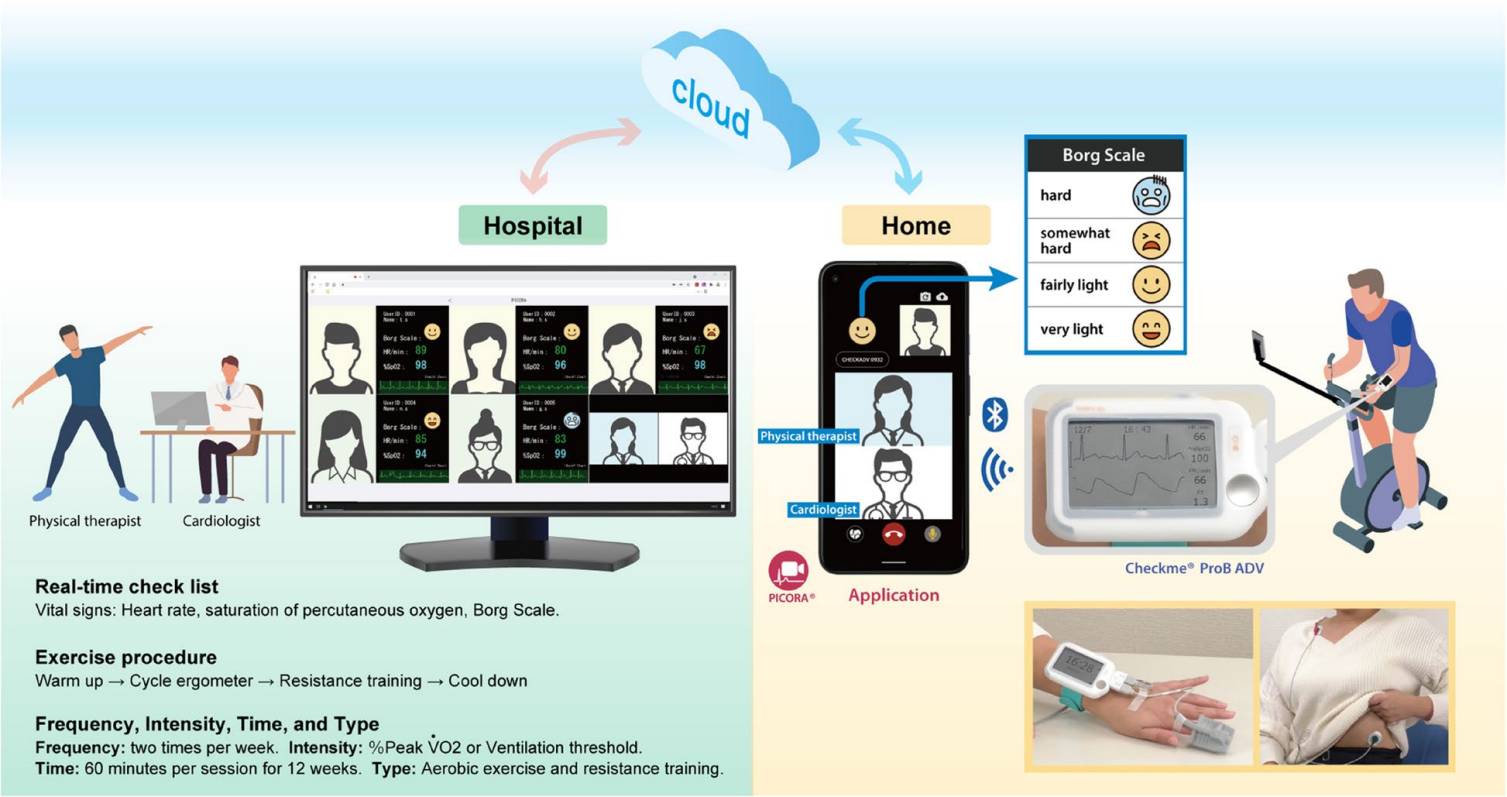
Real-time remote cardiac rehabilitation programme and ECG monitoring system. The patient wears the ECG measurement device and opens the application. A medical interview is conducted in the hospital, and the patient receives exercise instructions. ECG, electrocardiogram; VO_2_, oxygen uptake

Patients in the remote CR programme used the PICORA™ mobile phone application (SAN-EI Medisys, Inc.) using a smartphone or tablet device at a predetermined time. Subsequently, an ECG was obtained, and percutaneous oxygen saturation was measured using check me™ (SAN-EI Medisys, Inc.)

The data were transmitted to a smartphone or tablet device using Bluetooth® wireless technology (Bluetooth is a registered trademark of the Bluetooth Special Interest Group, Inc.).

Cardiologists and physical therapists provided guidance on exercise and education. A cardiologist evaluated blood pressure, heart rate, weight, and respiratory distress and checked for any chest symptoms and shortness of breath, particularly before rehabilitation training. The cardiologist and physical therapists monitored the ECG for any findings of atrial fibrillation, ventricular tachycardia/fibrillation, or premature ventricular contractions. The presence of any of these findings would have resulted in the patient to undergo a detailed examination at a hospital.

### Cardiac rehabilitation

The exercise training prescription adopted the FITT (frequency, intensity, time duration, and type of exercise) model. The following procedures were used in hospital-based CR and remote CR: (1) preparatory exercises, (2) using a bicycle ergometer or treadmill walking, (3) resistance training of the upper and lower extremities, and (4) organisational exercises. In addition, the exercise frequency was twice weekly, and the intensity of the bicycle ergometer was 40–60% of aerobic VT or PeakVO_2_ exercise intensity plus 50–60% of 1RM exercise intensity for resistance training. Resistance training was performed using 10–15 repetitions in three sets, maintaining a rate of perceived exertion (RPE) of 11–14 [23]. In the hospital-based CR, eight types of exercises were performed using leg presses and an elastic band (upper and lower extremity exercises). In addition, eight types of exercises were performed during the remote CR, including squats, heel raises, and elastic band use (upper and lower extremity exercises). The exercise intensity was gradually increased using the RPE. Using the bicycle ergometer or treadmill walking and preparatory and organisational exercises took place for over 1 h. The exercise types comprised preparatory, aerobic, resistance training, and organisational exercises. CR also included providing nutritional and exercise guidance in a timely manner. The number of unsupervised exercise sessions was calculated according to metabolic equivalents of tasks (METs).

### Quasi-randomisation and sample size

The assignment of medical record IDs was performed randomly. The allocation of patients was based on the last digit of their medical record numbers (IDs). Patients with even-numbered IDs were allocated to the hospital-based CR group, whereas those with odd-numbered IDs were assigned to the remote CR group. The allocation method was blinded until it was completed. Assignment and enrolment were performed by the authors.

The minimum sample size for the pilot study was determined as 12 per group [24]. Kieser and Wassimer applied an 80% upper confidence limit approach to sample size calculations and stated that a pilot study sample size of 20 corresponds to standardised effect sizes of 0.4 and 0.7 [25]. Furthermore, previous studies have used remote CR settings of 5–6 [26], 20 [27], and 60 cases [28] in each group. Considering these factors, this pilot study was designed to include 20 cases in each group.

### Statistical analysis

The baseline characteristics and outcomes were summarised, and continuous variables presented as mean ± SD. The Δ values of the outcome measures are expressed as percentage changes before and after the programme. First, the outcome variable was calculated as change from the baseline. We analysed whether the distribution was normal or non-normal. The numerical variables of independent samples were compared between the two groups. Nominal scales were compared using the χ^2^-square test. Differences between baseline and post-trial measurements were evaluated using a paired-samples *t*-test.

For the variables significantly (*p* < 0.05) related to Δ_peak_ VO_2_, a forward stepwise multiple regression analysis was performed using Δ_peak_ VO_2_ as the dependent variable. Statistical significance was set at *p* < 0.05. All statistical analyses were performed using IBM SPSS Statistics version 28 (IBM Corp., Armonk, NY, USA).

## Results

**Fig. 2:**
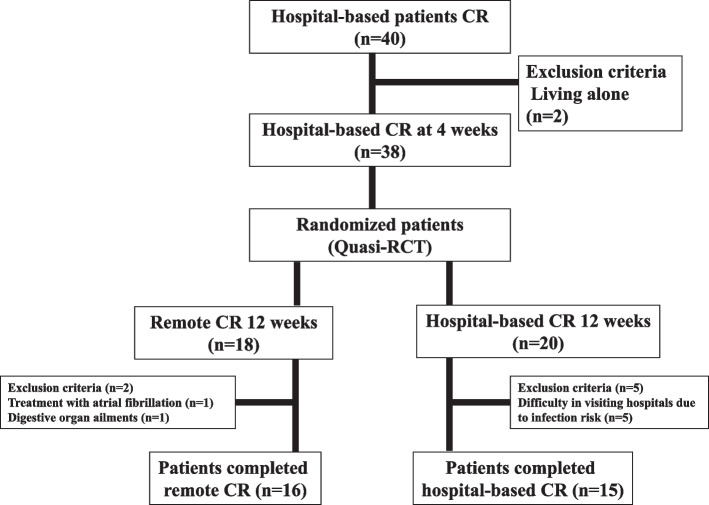
Flowchart of the study design. Overall, 38 patients, excluding those who lived alone, were assigned to quasi-randomisation. Twelve weeks of CR were performed. The 31 patients who completed the study were analysed (remote-based CR, *n* = 16; hospital-based CR, *n* = 15). CR, cardiac rehabilitation; Quasi-RCT, quasi-randomised controlled trial

We identified 38 patients with CVDs who were eligible for hospital-based CR after excluding two individuals who lived alone (Fig. 2). Sixteen patients (mean age: 72.2 ± 10.4 years) with a mean brain natriuretic peptide (BNP) level of 119.4 ± 111.3 pg/mL completed the remote CR programme. Moreover, 15 patients completed hospital-based CR (mean age: 77.3 ± 4.8 years, mean BNP level: 115.8 ± 115.0 pg/mL). Seven patients were excluded because of other health complications (*n* = 2), concerns about infectious diseases, and the inability to attend hospital-based CR (*n* = 5). The baseline characteristics of the 31 participants are summarised in Table 1. No major complications occurred during training. In this study, we confirmed that exercise intensity (METs) equivalent to that for CR was applied on other days. Remote CR was performed approximately 1.56 ± 0.81 times/week; in contrast, hospital-based CR was performed approximately 0.33 ± 0.48 times/week (*p* < 0.01).

**Table 1:** Baseline characteristics of the study population

	**Remote CR**	**Hospital CR**
	**Mean ± SD**	**Mean ± SD**
Disease HF/AMI for PCI/Post CS	13 / 2 / 1	14 / 0 / 1
COVID-19 vaccine inoculated (%)/unvaccinated (%)	10 (62)/6 (38)	8 (53)/7 (47)
Distance (between home and hospital) (km)	6.77 ± 7.1	5.51 ± 7.7
Dropout (%)	2/18 (11)	5/20 (25)
Men (%)/women (%)	7 (43)/9 (57)	12 (80)/3 (20)
Age	72.2 ± 10.4	77.3 ± 4.8
Height (cm)	156.1 ± 9.9	156.9 ± 8.5
Weight (kg)	58.8 ± 9.7	58.4 ± 11.3
BMI	24.1 ± 3.4	23.6 ± 3.2
**CPX**		
Peak VO_2_ (mL/min/kg)	12.0 ± 2.7	11.4 ± 2.8
VT V-slope (mL/min/kg)	8.9 ± 2.0	9.1 ± 2.5
VT trend (mL/min/kg)	8.6 ± 1.6	8.5 ± 2.2
Peak watt	59.7 ± 17.8	56.3 ± 23.7
Peak R	1.1 ± 0.1	1.1 ± 0.1
VE vs VCO_2_	31.6 ± 6.4	33.5 ± 6.9
R Knee joint extension muscle strength (kgf)	21.1 ± 9.2	20.3 ± 6.4
6MWD (m)	327.5 ± 105.9	298.3 ± 99.7
R Grip strength (kg)	24.1 ± 7.8	21.4 ± 6.4
**Echocardiography**		
LVEF (%)	59.2 ± 7.2	58.0 ± 7.6
AOD (mm)	35.3 ± 4.8	35.5 ± 5.7
LAD (mm)	41.7 ± 6.7	39.9 ± 5.2
IVSd (mm)	11.1 ± 2.7	11.8 ± 3.2
IVSs (mm)	12.6 ± 2.5	13.0 ± 2.5
LVDd (mm)	47.8 ± 7.6	47.2 ± 7.9
LVDs (mm)	33.1 ± 7.6	33.3 ± 8.5
SV (ml)	63.2 ± 20.2	60.0 ± 22.5
**Drawing blood**		
BNP (pg/ml)	119.4 ± 111.3	115.8 ± 115
eGFR (ml/min/1.73m^2^)	54.8 ± 22.2	59.8 ± 18.5
BUN (mg/dl)	21.9 ± 9.8	18.7 ± 7.8
Cr (mg/dl)	1.4 ± 1.9	0.9 ± 0.3
Albumin (g/dl)	4.0 ± 0.5	4.0 ± 0.5
**WHOQOL-BREF**		
Physical health (point)	20.3 ± 4.5	20.1 ± 4.7
Psychological health (point)	17.1 ± 3.4	16.3 ± 3.5
Social relationships (point)	10.0 ± 1.9	8.2 ± 3.0
Environment (point)	25.5 ± 4.1	22.5 ± 5.2
Total (point)	77.4 ± 12.9	71.7 ± 16.4
**WHODAS2.0-J**		
Cognition (point)	10.0 ± 11.2	16.7 ± 30.0
Mobility (point)	21.4 ± 21.2	17.0 ± 20.2
Self-care (point)	13.3 ± 24.6	12.8 ± 19.7
Social interactions (point)	22.3 ± 21.2	22.7 ± 20.2
Life activities (point)	36.8 ± 27.0	26.0 ± 25.8
Participation (point)	28.6 ± 22.7	24.4 ± 25.7
Total (point)	21.6 ± 14.3	19.3 ± 21.8

Preintervention physical and mental function, echocardiographic ultrasound, and blood test results

*BMI* body mass index, *CPX* cardiopulmonary exercise test, *Peak VO*_*2*_ maximum oxygen uptake_;_ V-slope method: rising point of VCO_2_ relative to VO; VEQ method: Point of increase in the ventilatory equivalent of oxygen (VE/VO_2_) without a concurrent increase in the ventilatory equivalent of carbon dioxide (VE/VCO_2_), *VE vs VCO*_*2*_ minute ventilation vs carbon dioxide production, *6MWD* 6 min walk distance, *LVEF* left ventricular ejection fraction, *AOD* aortic root diameter, *LAD* left atrial dimension, *IVSd* diastolic interventricular septum, *IVSs* systolic interventricular septum, *LVDd* left ventricular end-diastolic dimension, *LVDs* left ventricular internal dimension in systole, *SV* left ventricular stroke volume, *BNP* brain (B type) natriuretic peptide, *Egfr* estimated glomerular filtration rate, *BUN* blood urea nitrogen, *Cr* creatinine, *HF* heart failure, *AMI* acute myocardial infarction, *PCI* percutaneous coronary intervention, *CS* cardiac surgery

There were no significant differences in the background characteristics of the patients (Table 1). No adverse events were observed during the study. The continuation rate was 88% for remote CR and 75% for hospital-based CR (*p* = 0.24). The WHOQOL-BREF and WHODAS2.0-J total scores for remote CR were 77.4 ± 12.9 and 21.6 ± 14.3 points, respectively.

The physical function of the remote CR group significantly differed from that of the hospital-based CR group (Δ_peak_ VO_2_: remote CR, 2.8 ± 3.0 mL·min^−1^·kg^−1^ versus hospital-based CR, 0.84 ± 1.8 mL·min^−1^·kg^−1^; *p* < 0.05). The WHOQOL-BREF scores did not differ between the remote and hospital-based CR groups. Furthermore, the WHODAS2.0-J total scores did not significantly differ between the groups (ΔWHODAS2.0-J: remote CR, -6.8 ± 16.7 points versus hospital-based CR, 2.1 ± 8.0 points; *p* = 0.06; Table 2).

**Table 2:** Between group treatment effects (Δ)

	**Remote CR**	**Hospital CR**	
	**Mean ± SD**	**Mean ± SD**	***p*-value**
ΔPeak VO_2_ (ml/min/kg)	2.8 ± 3.0	0.84 ± 1.82	*p* < 0.02
ΔVT trend (ml/min/kg)	1.44 ± 1.77	0.95 ± 1.65	*p* = 0.17
ΔR Knee joint extension muscle strength (kgf)	9.1 ± 4.1	5.6 ± 4.6	*p* < 0.03
Δ6MWD (m)	94.1 ± 88.8	36.6 ± 41.3	*p* < 0.02
**WHOQOL-BREF**			
ΔPhysical Health (point)	5.6 ± 5.7	3.0 ± 4.1	*p* = 0.15
ΔPsychological (point)	4.6 ± 5.5	2.67 ± 3.9	*p* = 0.25
ΔSocial relationships (point)	0.6 ± 1.6	2.67 ± 2.6	*p* = 0.19
ΔEnvironment (point)	0.6 ± 1.6	1.67 ± 2.6	*p* = 0.19
ΔTotal (point)	18.3 ± 14.7	10.53 ± 14.3	*p* = 0.14
**WHODAS2.0-J**			
ΔCognition (point)	− 3.1 ± 9.9	3.6 ± 13.4	*p* = 0.11
ΔMobility (point)	− 5.0 ± 24.2	5.4 ± 17.0	*p* = 0.17
ΔSelf-care (point)	− 12.5 ± 24.9	− 1.3 ± 8.3	*p* = 0.10
ΔSocial interactions (point)	− 6.7 ± 18.5	1.6 ± 10.9	*p* = 0.13
ΔLife activities (point)	− 15.0 ± 34.2	1.3 ± 10.6	*p* = 0.08
ΔParticipation (point)	− 15.1 ± 24.9	− 0.2 ± 11.8	*p* < 0.05
ΔTotal (point)	− 6.8 ± 16.7	2.1 ± 8.0	*p* = 0.06

The difference in the treatment effect between the two groups is expressed as Δ

After remote CR, the peak VO_2_ (pre-CR: 12.0 ± 2.7 mL·min^−1^·kg^−1^; post-CR: 14.9 ± 3.9 mL·min^−1^·kg^−1^; *p* < 0.05) and WHOQOL-BREF score (pre-CR: 77.4 ± 12.8 points; post-CR: 93.9 ± 12.9 points; *p* < 0.001) were significantly higher and the WHODAS2.0-J score was significantly lower (pre-CR: 21.6 ± 14.3 points; post-CR: 12.3 ± 7.4 points; *p* < 0.05; Table 3) than those at baseline. No adverse events were observed.

**Table 3:** Therapeutic effects on physical and mental functions before and after the different cardiac rehabilitation programmes

	**Pre-Remote CR**	**Post-Remote CR**		**Pre-Hospital CR**	**Post-Hospital CR**	
	**Mean ± SD**	**Mean ± SD**	***p*-value**	**Mean ± SD**	**Mean ± SD**	***p*-value**
**CPX**						
Peak VO_2_ (ml/min/kg)	12.0 ± 2.7	14.9 ± 3.9	*p* < 0.05	11.4 ± 2.8	12.2 ± 2.9	*p* = 0.09
VT V-slope (ml/min/kg)	8.9 ± 2.0	10.0 ± 2.5	*p* < 0.05	9.1 ± 2.5	9.5 ± 2.5	*p* = 0.40
VT trend (m//min/kg)	8.6 ± 1.6	10.0 ± 2.6	*p* < 0.05	8.5 ± 2.2	9.4 ± 2.5	*p* = 0.32
Peak watt	59.7 ± 17.8	78.0 ± 20.7	*p* < 0.001	56.3 ± 23.7	61.1 ± 21.6	*p* = 0.07
Peak R	1.1 ± 0.1	1.2 ± 0.1	*p* = 0.22	1.1 ± 0.1	1.08 ± 0.08	*p* = 0.53
VE vs VCO_2_	31.6 ± 6.4	32.4 ± 5.3	*p* = 0.97	33.5 ± 6.9	40.8 ± 31.4	*p* = 0.33
R Knee joint extension muscle strength (kgf)	21.1 ± 9.2	30.2 ± 10.3	*p* < 0.001	20.3 ± 6.4	25.9 ± 6.5	*p* < 0.001
6MWD (m)	327.5 ± 105.9	413.1 ± 110	*p* < 0.05	298.3 ± 99.7	335 ± 94.2	*p* < 0.05
R Grip strength (kg)	24.1 ± 7.8	26.5 ± 8.2	*p* < 0.05	21.4 ± 6.4	22.3 ± 6.8	*p* = 0.13
**WHOQOL-BREF**						
Physical Health (point)	20.3 ± 4.5	25.2 ± 3.7	*p* < 0.001	20.1 ± 4.7	24.1 ± 5.8	*p* = 0.21
Psychological health (point)	17.1 ± 3.4	21.0 ± 3.1	*p* < 0.05	16.3 ± 3.5	20.1 ± 3.8	*p* = 0.20
Social relationships (point)	10.0 ± 1.9	10.6 ± 1.8	*p* = 0.13	8.2 ± 3.0	10.0 ± 2.3	*p* < 0.05
Environment (point)	25.5 ± 4.1	29.6 ± 4.0	*p* < 0.05	22.5 ± 5.2	27.8 ± 6.6	*p* < 0.001
Total (point)	77.4 ± 12.9	93.9 ± 12.9	*p* < 0.001	71.7 ± 16.4	88.0 ± 18.7	*p* < 0.001
**WHODAS2.0-J**						
Cognition (point)	10.0 ± 11.25	6.8 ± 10.6	*p* = 0.23	16.7 ± 30.0	20.0 ± 28.4	*p* = 0.30
Mobility (point)	21.4 ± 21.2	16.4 ± 15.4	*p* = 0.41	17.0 ± 20.2	22.4 ± 24.0	*p* = 0.23
Self-care (point)	13.1 ± 24.4	0.63 ± 2.5	*p* = 0.06	12.8 ± 19.7	11.4 ± 19.9	*p* = 0.54
Social interactions (point)	22.3 ± 21.2	15.5 ± 14.8	*p* = 0.16	22.7 ± 20.2	24.4 ± 20.0	*p* = 0.56
Life activities (point)	36.8 ± 27.0	21.8 ± 17.2	*P* = 0.10	26.0 ± 25.8	27.3 ± 28.1	*p* = 0.63
Participation (point)	28.6 ± 22.7	13.5 ± 11.8	*p* < 0.05	24.4 ± 25.7	24.1 ± 29.1	*p* = 0.93
Total (point)	21.6 ± 14.3	12.3 ± 7.4	*p* < 0.05	19.3 ± 21.8	21.5 ± 23.0	*p* = 0.31

Univariate correlation analysis showed a significant positive correlation between Δpeak VO_2_ and baseline self-care (*r* = 0.38, *p* < 0.05), baseline social interactions (*r* = 0.46, *p* < 0.05), and life activities (*r* = 0.42, *p* < 0.05).

Remote CR was significantly positively correlated with baseline mobility (*r* = 0.60, *p* < 0.05), self-care (*r* = 0.59, *p* < 0.05), and social interaction level (*r* = 0.80, *p* < 0.01). In addition, hospital CR was significantly negatively correlated with baseline mobility (*r* = -0.52, *p* < 0.05) (Table 4).

**Table 4:** Univariate correlation coefficients for the Δpeak VO_2_ with WHOQOL-BREF and WHODAS2.0-J

	Total (*n* = 31)	Remote (*n* = 16)	Hospital (*n* = 15)
	r	r	r
**WHOQOL-BREF**			
Physical Health	− 0.236	− 0.579	0.424
Psychological	− 0.306	− 0.608	0.241
Social relationships	0.2	− 0.242	0.422
Environment	− 0.06	− 0.516	0.21
Total	− 0.141	− 0.594	0.367
**WHODAS2.0-J**			
Cognition	− 0.16	0.17	− 0.39
Mobility	0.22	0.60 *	− 0.52 *
Self-care	0.38 *	0.59 *	− 0.01
Social interactions	0.46 *	0.80 **	− 0.11
Life activities	0.42 *	0.60 *	− 0.24
Participation	0.12	0.24	− 0.11
Total	0.24	0.67 *	− 0.22

^*^p < 0.05. ** *p* < 0.01. WHOQOL-BREF showed no correlation. In contrast, WHODAS2.0-J showed a correlation

Forward multiple stepwise regression analysis using overall data showed that the intervention method (β = 0.339, *p* < 0.05), baseline cognition (β =  − 0.424, *p* < 0.05), and social interaction level (β = 0.658, *p* < 0.01; WHODAS2.0-J) were significant independent contributors to Δpeak VO_2_ (R^2^ = 0.48, F = 8.13, *p* < 0.01) (Table 5).

**Table 5:** Results of the stepwise multiple regression analysis

Dependent variable	Independent variable	β-value	B-value	*p*-value	R2	F-value (*p*-value)
**Δ Peak VO**_**2**_	Remote-based and hospital-based	0.339	1.783	*p* < 0.05	0.48	8.13(*p* < 0.01)
	**WHO DAS 2.0-J**					
	Cognition	-0.424	-0.258	*p* < 0.05		
	Social interactions	0.658	0.718	*p* < 0.01		

## Discussion

In this study, we compared the effectiveness of real-time remote CR with that of hospital-based CR for improving physical function, subjective satisfaction, and objective performance (i.e., activity limitations and participation restrictions). The physical functioning of the remote CR group markedly improved compared with that of the hospital-based CR group.

Data obtained from several studies on home-based CR suggest that it yields results comparable to those of hospital-based programmes [6, 29]. Research on remote-based CR has focused on non-supervised types [6, 29], and there are few studies on real-time monitoring types [9]. Furthermore, there is no established system to detect adverse events (e.g., arrhythmias and chest symptoms). In addition, there are challenges in terms of cost-effectiveness and other factors. According to Ralph et al., real-time remote CR is an effective and cost-effective treatment modality [12]. Remote CR, including the unmonitored type, showed no significant difference in total mortality and exercise capacity [10]. However, these previous studies were reported before the COVID-19 pandemic. Therefore, the physical functions as well as subjective satisfaction and objective performance reported in these studies could vary accordingly.

COVID-19 risk, close contact, and infection anxiety were associated with the use of public transportation in a previous study [30]. Therefore, these quality-of-life domains might have been adversely affected when this study was performed, which was during the COVID-19 pandemic.

COVID-19 is caused by the severe acute respiratory syndrome coronavirus 2, which is easily transmitted through the eyes, tongue, and nasal passages [31].

Patients with CVD are at risk of severe disease if they contract COVID-19 [32, 33]. There was a high degree of constraint in terms of outdoor activity among older adults during this period because of the fear of infection. Even in our cohort, five (25%) patients in the hospital-based CR group were excluded because they refrained from going out owing to concerns about infection.

Two participants in the remote CR group were excluded as one of them required treatment for atrial fibrillation and another patient developed a digestive system disease. Therefore, remote CR can eliminate the concerns regarding infectious diseases. No adverse events were observed during the study. In remote CR, real-time management of ECGs and blood pressure measurements during exercises are pertinent challenges [34]. The remote ECG management device used in this study can be used to collectively manage ECG and oxygen saturation changes using the Borg scale in real time. Therefore, it is useful for the early detection of physical abnormalities in patients with CVDs. However, remote CR presents safety challenges. Hospital-based CR with direct monitoring by medical personnel facilitates a timely and appropriate response to emergencies. In contrast, in remote CR, responses to emergency situations are provided by the family members. Therefore, patient selection and emergency response methods should be carefully considered. In this study, peak VO_2_ (hospital-based CR: 11.4 ± 2.8 mL·min^−1^·kg^−1^, remote CR: 12.0 ± 2.7 mL·min^−1^·kg^−1^) in association with both hospital-based and remote CR exceeded a mean of 10 mL·min^−1^·kg^−1^. Remote CR in frail patients or those with sarcopenia is associated with adverse events and relative risks, such as falls. We had prepared for such adverse events by explaining cardiopulmonary resuscitation and emergency response techniques by providing DVDs to the patients' families. In addition, new-onset atrial fibrillation could be identified by checking the ECG.

Many studies have been conducted to evaluate the improvements in physical function due to CR. Restrictions on outings due to the COVID-19 pandemic in Japan have increased the risk of frailty and resulted in weight gain in patients undergoing convalescent CR [35]. This study showed a considerable improvement in the remote CR group.

CR requires comprehensive interventions such as counselling, exercise, and education [36]. In this study, remote CR and walk-in CR were set twice weekly. Therefore, development of positive exercise habits (on other days) was very important. The extent to which exercise intensity (METs) and duration of exercise were set up to be equivalent to CR was evaluated in this study. The results revealed that remote CR METs were performed for an average of 1.56 ± 0.81 times/week and hospital-based CR METs were performed for an average of 0.33 ± 0.48 times/week (*p* < 0.01). Equivalent training could be performed at home on days other than exercise intervention days in the remote CR group. This might have accounted for the difference in the changes in physical function between the two groups in the current study. Remote CR allows similar training, making it easier to be incorporated into daily life. In contrast, in the outpatient setting, the exercise setting (METs) had to be outdoors, which diverges from the exercise items that are administered during CR. Furthermore, the frequency of exercise performed depended on the season and weather conditions. In other words, creating a CR environment within the home is important.

Educational guidance in this study included not only dietary guidance but also exercise guidance in the home environment. We believe that these exercises can be performed similar to remote CR, which would result in more marked improvements in outcomes. Improving quality of life is an important goal of CR, and during the COVID-19 pandemic, health literacy and health-related quality of life have declined [31].

The WHODAS2.0-J is currently the only measure based on the International Classification of Functioning, Disability and Health biopsychosocial model [37]. The WHODAS2.0-J asks what a person “does” in a particular domain, while the WHOQOL-BREF asks what the person “feels” in that domain. However, there are no reports of remote-based CR using the WHODAS2.0-J and WHOQOL-BREF.

Therefore, we used the WHOQOL-BREF and WHODAS2.0-J for a comprehensive assessment of quality of life. The ΔWHOQOL-BREF scores did not differ between the remote and hospital-based CR groups. Moreover, the ΔWHODAS2.0-J scores did not significantly differ between the groups.

Real-time remote CR revealed extensively improved participation based on the WHODAS2.0-J scores. The patients were asked how their situations and the people around them made it difficult for them to participate in social activities; it also included questions about the impact of health conditions on quality of life.

With regard to participation, remote CR was more effective than hospital-based CR. Further, we can conclude that remote CR is equivalent to hospital-based CR in terms of performance in the other domains, including cognition, mobility, self-care, social interaction, and life activities.

In addition, objective performance was markedly improved with remote CR. We examined patients in terms of cognition and social interaction levels using the WHODAS2.0-J and found that the results contributed extensively to Δpeak VO_2_. Remote CR is an effective treatment modality to improve quality of life. In remote CR, it is important to assess not only physical function but also objective performance and subjective satisfaction.

Finally, four issues were identified regarding the feasibility of remote CR: (1) response to equipment failure, (2) cost of medical equipment, (3) transition period from hospital-based CR to remote CR, and (4) emergency response. These are discussed in detail below.

1. Response to equipment failure

It was established beforehand in this investigation that the means of communication were stable. However, if the communication means were not adequate or feasible, it would have been difficult to complete the study. This can be affected by social infrastructure facilities and is highly likely to vary between nations; this is an issue to be addressed in the future.

(2) Cost of medical equipment

In this study, medical equipment was provided to each patient and was used for 3 months. The cost of the equipment per patient was higher than that for the hospital CR as equipment was provided to each patient in the hospital. The challenge remains as to how to reduce the cost of medical devices while increasing convenience for patients.

(3) The transition from hospital-based CR to remote CR

In this study, the patients underwent hospital-based CR sessions in the first 4 weeks to confirm that there were no adverse events. However, there are many instances in which a 4-week hospital-based CR is also difficult. Therefore, the decision regarding the timing of the transition to remote CR is a major issue for future implementation.

(4) Emergency response

ECG monitoring can be used to quickly confirm changes, which is a major point in this study. If a patient meets the criteria for discontinuation, the treatment should be terminated immediately, and family support should be sought. We factored this in and accordingly prepared an emergency response manual in advance. We believe that each medical institution should prepare an adequate emergency response manual and discuss it with patients and family members prior to implementation.

### Study limitations

As the study was conducted during the COVID-19 pandemic, the number of participants was very limited. Patients eligible for hospital-based CR and those who lived alone or had severe CVDs were excluded. This study was designed as a quasi-randomised controlled trial for safety reasons. As no adverse events were observed in this study, a randomised controlled trial is needed. Furthermore, this was a single-centre study; a multicentre collaborative study should be considered in the future. In addition, the quasi-randomisation-controlled trial setting limits the potential generalisation of the findings.

Furthermore, the present study was conducted on a small sample size. Based on the results of this study, future studies should include larger sample sizes. In the future, comprehensive research is needed to clarify the efficacy of remote CR in severely ill patients and patients living alone. Future studies are also warranted for further risk stratification according to illness severity.

## Conclusions

Remote CR considerably improved the physical function and objective performance of patients with CVDs. Remote CR can be an effective treatment modality for stable patients who cannot visit hospitals during the COVID-19 pandemic. Further research with larger sample sizes and modifications in the methodology based on the findings of this study is warranted to formulate more evidence-based conclusions.

## Data Availability

The datasets used and/or analysed during the current study are available from the corresponding author on reasonable request.

## Abbreviations

CR: Cardiac rehabilitation
CVD: Cardiovascular disease
COVID-19: Coronavirus disease 2019
CPX: Cardiopulmonary exercise test
MET: Metabolic equivalent of task
VO_2_: Oxygen uptake
VT: Ventilation threshold
WHOQOL-BREF: Japanese version of the World Health Organization Quality of Life Instrument
WHODAS2.0-J: Japanese version of the World Health Organization Disability Assessment Schedule, 36-item version, self-administered
WHO-QOL: World Health Organization Quality of Life
